# Peripheral quantitative computed tomography (pQCT) for the assessment of bone strength in most of bone affecting conditions in developmental age: a review

**DOI:** 10.1186/s13052-016-0297-9

**Published:** 2016-09-26

**Authors:** Stefano Stagi, Loredana Cavalli, Tiziana Cavalli, Maurizio de Martino, Maria Luisa Brandi

**Affiliations:** 1 Health Sciences Department, University of Florence, Anna Meyer Children’s University Hospital, viale Pieraccini 24, 50139 Florence, Italy; 2 Department of Surgery and Translational Medicine, Endocrinology Unit, University of Florence, Florence, Italy; 3 Department of Surgery and Translational Medicine, Emergency and Digestive Surgery with Oncological and Functional Address Unit, University of Florence, Florence, Italy

**Keywords:** Bone mineralization, Peripheral quantitative computed tomography (pQCT), Bone mass, Bone mineral density, Muscle area, Fat area, Osteoporosis

## Abstract

Peripheral quantitative computed tomography provides an automatical scan analysis of trabecular and cortical bone compartments, calculating not only their bone mineral density (BMD), but also bone geometrical parameters, such as marrow and cortical Cross-Sectional Area (CSA), Cortical Thickness (CoTh), both periosteal and endosteal circumference, as well as biomechanical parameters like Cross-Sectional Moment of Inertia (CSMI), a measure of bending, polar moment of inertia, indicating bone strength in torsion, and Strength Strain Index (SSI). Also CSA of muscle and fat can be extracted. Muscles, which are thought to stimulate bones to adapt their geometry and mineral content, are determinant to preserve or increase bone strength; thus, pQCT provides an evaluation of the functional ‘muscle-bone unit’, defined as BMC/muscle CSA ratio. This functional approach to bone densitometry can establish if bone strength is normally adapted to the muscle force, and if muscle force is adequate for body size, providing more detailed insights to targeted strategies for the prevention and treatment of bone fragility. The present paper offers an extensive review of technical features of pQCT and its possible clinical application in the diagnostic of bone status as well as in the monitoring of the skeleton’s health follow-up.

## Background

Osteoporosis (OP) may be recognized as one of the major public health problems, growing both in paediatrics and adulthood, irrespective of the gender [1–3]. It is a skeletal disorder characterized by compromised bone strength because of decreased bone mineral density (BMD) and altered trabecular bone architecture [4]. The Dual Energy X-ray Absorptiometry scan (DXA) is recommended as the first clinical tool for diagnosing OP [5, 6]. However, due to difficulties in the detection of relatively small changes along longitudinal studies, DXA scans is not useful in monitoring OP treatment or prevention on a routine basis.

Although, high-resolution peripheral quantitative computed tomography (HR-pQCT), which has been shown to have the same reproducibility of BMD assessed by DXA at the forearm [7] and a good accuracy [8], appears to be more promising because it allows to determine a direct quantification of bone structure, other than BMD, with resolutions below 100 μm [8]. Changes in trabecular morphology are reported to lead to a disproportionate decrease in bone strength [9], therefore microstructural information should be included in the analysis to accurately predict individual mechanical bone properties [10–13]. However, the relative importance of bone density and architecture in the aetiology of bone fractures is still poorly understood.

In contrast, peripheral Quantitative Computed Tomography (pQCT) enables bone parameters of the peripheral skeleton (e.g. radius, tibia and femur) to be investigated in details. While providing low radiation doses, pQCT enables calculation of volumetric BMD (vBMD), instead of the projected areal BMD obtained by DXA. Furthermore, using pQCT, trabecular and cortical bone parameters can be analyzed separately, thus allowing the examination of bone distribution, architecture and geometry at fracture-prone sites. The use of pQCT technology in one of our studies [14] provided a more comprehensive assessment of the effects of Juvenile Idiopathic Arthritis (JIA) on overall bone size and strength as well as the delineation of trabecular and cortical bone characteristics and their relationship to muscle mass.

In this paper, a review of recent literature describing pQCT technique, and its use for assessing bone strength in different conditions has been reported.

### Peripheral quantitative computed tomography

**Fig. 1:**
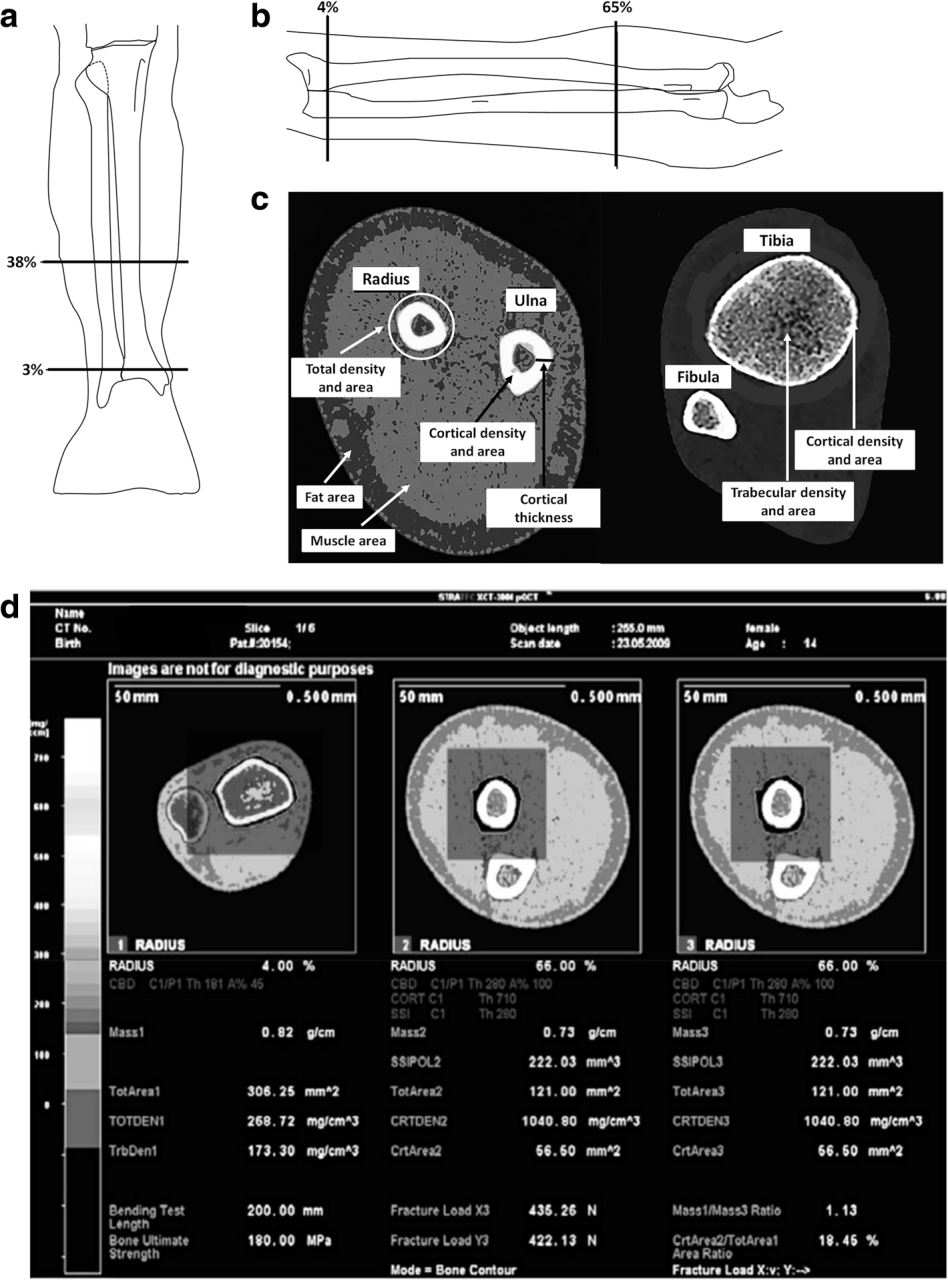
Typical tibia (**a**) and radius (**b**) pQCT, with scan location indicated as percent of the length of the respective bones. **c** Scan section for tibia and radius obtained by pQCT in a normal-weight girl. The image allows identifying some of the main parameters obtained, such as the fat cross-sectional area (CSA) (fatCSA) and the muscle CSA (muscleCSA). **d** Example of a forearm pQCT scan.in an osteoporotic 14-year-old patient

Quantitative computed tomography (QCT) is a three-dimensional non-projection technique to quantify BMD in the spine, proximal femur, forearm, and tibia with a number of advantages to other densitometry techniques: cortical and trabecular bone can be separated, trabecular volumes of interest (VOI) are largely independent of spine degenerative changes, and 3D geometric parameters can be determined. PQCT is also increasingly used to study the interaction between muscle and bone systems [15, 16], being able to benefit from high-quality, accurate imaging and morphological assessment which allow an accurate diagnosis and treatment of a musculoskeletal diseases spectrum [17] (Fig. 1).

PQCT at the forearm was introduced shortly after computed tomography (CT) for medical imaging and several years before the development of spinal QCT, as a volumetric extension to Cameron’s projection technique for bone mineral measurements [18]. For a long period the technical implementations of QCT by using clinical CT scanners, and of pQCT with smaller dedicated forearm scanners, remained virtually unchanged. A number of single-slices, e.g. one slice for each lumbar vertebra L1-L4, are scanned; then, for each acquired slice, a CT image is reconstructed in which dedicated regions of interest (ROI) for trabecular and cortical compartments are analyzed. BMD values are then derived, either from a calibration procedure using an in-scan calibration phantom measured below the patient, or from stored calibration values obtained before the patient scan.

Renewed interest in QCT/pQCT has been increased by several reasons: a) the rapid progress in CT technology since the introduction of spiral CT, which enables the acquisition of volumetric scans of skeletal sites other than spine, such as femur; b) the ability to measure cortical and trabecular bone separately, c) to provide information of bone geometry and trabecular structure, and d) to quantify BMD independently from bone size, unlike DXA [19]. In fact, areal density (aBMD or BMDa) as measured by DXA is typically better correlated with body weight and height than BMD as measured by QCT, or a volumetric estimation of bone mineral density (BMAD), obtained by dividing DXA aBMD by the square root of bone area. Finally, it has been reported in several pharmaceutical trials that increases in aBMD, as measured by DXA, do not entirely explain the observed reduction in fracture risk, highlighting some challenges in monitoring treatment with DXA.

### Image generation in CT

The image generation in CT is a two-step process [19]. First, a survey radiograph, called a *scout scan* or *topogram*, is acquired in order to locate the scan range for the tomographic images. Newer systems equipped with multiple rows of detectors acquiring up to 64 slices simultaneously are designated as *multi-slice CT* (MSCT). Scan times for MSCT are typically below 10 s for the lumbar spine (LS) or the proximal femur. A further development in MSCT is the use of areal detectors, which acquire even larger volumes in one rotation, so that in many applications a spiral motion of the X-ray tube relative to the body is no longer necessary. This true volumetric data acquisition is used in a new dedicated forearm scanner, but has not yet been introduced in whole body (WB) CT scanners.

After acquisition, the second step of CT image generation is the tomographic reconstruction, which describes the mathematical process of calculating the image from the acquired data. It is important to separate these two steps, as the operator can select a variety of parameters for both that influence image quality.

### The forearm pQCT

Standard pQCT scanners work in step and scan mode. Some acquire single-slices, while others operate in multi-slice mode. Measurement locations are defined with respect to the length of the radius, measured from the radio-carpal joint surface to the olecranon. Typically, scan locations with single-slice CT scanners are distal sites (4 % of radius length), containing mainly trabecular bone, and a shaft location (15–65 % of radius length), consisting predominantly of cortical bone.

Multi-slice scanners use a distal site between 4 % and 10 % of the length of the radius and a shaft location. A decrease in slice thickness, e.g., from 2 mm to 1 mm, increases the geometrical resolution. Currently, only few types of pQCT scanners are in use. Most of the literature has been published for the XCT 2000 (Stratec Medizintechnik, Pforzheim, Germany) and its predecessors, the XCT 960 and the isotope based XCT 900. These are scan and step scanners that are predominantly used to determine BMD, bone mineral content (BMC), cortical width and volume, or cross-sectional area of the radius, to derive biomechanical parameters such as stress strain index (SSI) and moment of inertia [19].

### Patient positioning

As with any medical imaging device, the proper positioning of the patient is crucial to accuracy and reproducibility. The most common use of HR-pQCT in vivo is to scan the non-dominant radius and tibia. The scanner’s gantry, narrow and shallow, only allows the distal peripheral skeleton to be accommodated, then immobilized in a carbon fibershell. A scout view, essentially a two-dimensional x-ray scan, is obtained so that the operator can identify a precise region for the 3D measurement. The standard measurement protocol utilizes the following settings: an X-ray tube of 60kVp potential, X-ray tube current of 95 mA, matrix size of 1536 × 1536 and slice thickness and in-plane voxel size of 82 μm. While the reconstructed voxel size is 82 μm for the standard-patient HR-pQCT protocol, the actual spatial resolution of the image is approximately 130 μm near the centre of the field of view, and somewhat less off-centre (140–160 μm) [20, 21]. Consequently, structures less than 100 μm are not typically resolved from in vivo images. At each site, 110 computerized tomography slices are obtained and used to reproduce a 9.02 mm (radial or tibial length) three-dimensional image.

Daily and weekly quality control scans must be performed to identify drift, which can occur as a consequence of decreased X-ray emission (decay). The HR-pQCT single-scan effective dose is estimated to be 3 μSv [22]. Since exposure is additive, we recommend no more than three measures at a single site during an appointment, being the recommended radiation dose limit 50 μSv/year (Inter-national Commission on Radiological Protection). Since HR-pQCT uses a polychromatic X-ray source, it is subject to beam hardening, which can significantly impact geometric and density measures [23].

### Motion artefacts

As HR-pQCT has a high resolution and scan times are relatively long (3 min), any movement during the scan can result in artefact, compromising the accuracy and reproducibility of the images obtained [24]. Measures of micro-architecture are more sensitive to movement artefact compared with geometric or densitometry measures [25–28]. The best solution in case of motion is to re-scan the patient, since correction algorithms cannot be applied to the parameters themselves [24].

### Advantages of pQCT

The aBMD is commonly used to predict fractures at clinically important sites [29], influenced by several different skeletal parameters such as periosteal expansion, cortical BMD (BMDcort), cortical thickness (CoTh), trabecular number and trabecular thickness [30], all measures under distinct systems of biological and genetic control. Currently, it is unclear which of these bone parameters better identifies genetically determined phenotypes, or whether aBMD is more related to bone size or growth [31], or genetic features. Devices such as pQCT, which measure cross sections of predominantly cortical or trabecular bone, enable the different constituents of bone mass to be analyzed separately and offer advantages over DXA in terms of possible genotype/bone phenotype correlations. In recent genome-wide association studies, we examined how those genetic polymorphisms found to be associated with aBMD were also related to pQCT parameters, based on analysis of adolescents from the Avon Longitudinal Study of Parents and Children (ALSPAC) and young adult men from the ‘Gothenburg Osteoporosis and Obesity Determinants’ (GOOD) cohort [32]. In comparison to DXA, QCT or pQCT provide a more refined characterization of bone, including measures of vBMD, bone geometry (dimensions, area, and CoTh), and quantifies the distribution of mineral within cross-section, thus providing a better understanding of skeletal deficits associated with fracture risk.

Few studies of children with a forearm fracture have used QCT. A cross-sectional study of girls found no difference in trabecular or cortical vBMD of the distal radius; however, girls with a fracture had a narrower diameter of the radial metaphysis than girls without a fracture [33]. A longitudinal study of pubertal girls found that those who sustained an upper limb fracture had lower integral vBMD (cortical and trabecular bone), but a greater cross-sectional area of the radial metaphysis compared to controls, and these differences persisted throughout adolescence [34]. Lastly, among males aged 18, those with a previous forearm fracture had lower cortical and trabecular vBMD and lower cortical thickness, but no difference in periosteal circumference compared to non-fractured controls [35]. The discordant findings among studies make it difficult to draw conclusions about the relative importance of bone density and aspects of bone geometry for forearm fracture in children and adolescents [36].

Separate measurement of trabecular and cortical bone compartments provided by volumetric methods allows earlier detection of changes in bone mass in response to disease or therapy [36]. However, both pQCT and HR-pQCT are more sensitive to movement and require the subject to be still for nearly five minutes for optimal scans, which is difficult to obtain in very young children.

While pQCT is limited to assessment of peripheral sites, QCT, HR-QCT and pQCT have limited published pediatric specific reference data [36]. Therefore, it seems reasonable that DXA continues to be used to assess low bone mass in children, especially if adjustment for body size deficits are considered [36].

### Changes of bone characteristics during childhood and adolescence

During childhood and adolescence, skeletal development is characterized by increases in BMDtrab and BMDcort and cortical dimensions [37]. In healthy human subjects, bone mineral mass follows a trajectory from birth on to attain a maximal value, the so called peak bone mass (PBM), by the end of the second or the beginning of the third decade, according to both gender and skeletal sites examined [38]. Childhood and adolescence are critical periods for bone mass gain, since about 90 % of PBM is acquired before the age of 18, and a low PBM may increase fractures risk in adulthood [39]. Furthermore, this influence of pubertal timing on peak bone mass is predetermined before the onset of pubertal maturation [40]. Growth in infancy was reported to be associated with BMC in later life [41]. The risk of hip fracture in elderly results as related to early variation in height and weight growth [42, 43]. Glucocorticoid (GC) medications are highly effective and widely prescribed for the treatment of varied inflammatory conditions in children and adults. However, GC therapy in children is associated with multiple adverse side effects, including obesity, impaired linear growth, and increased fracture rates [44, 45]. Studies have consistently shown that GCs result in sustained reductions in bone formation because of decreased osteoblast differentiation and activity and increased osteoblast and osteocyte apoptosis [46].

Other than providing vBMD and cortical geometry, highly correlated with fracture load [47, 48], pQCT is able to measure muscle and fat cross-sectional area (CSA) [46].

### Soft-tissue measurements

Muscle and fat CSAs were estimated at the 66 % tibial and 25 % femoral pQCT scan sites. Combined muscle and fatCSA can be automatically calculated by subtracting CSAtot from the total image CSA. For the remaining area, the manufacturer’s threshold of 36 mg*cm^−3^ can be used to separate fatCSA from muscle CSA (muscleCSA or mCSA). Then a combination of filters allows extracting the total fat from the muscle. The bone-to-muscle ratio (BMR) is the ratio of cortical bone CSA (tibia + fibula) to muscleCSA, presented as a percentage. The fat-to-muscle ratio (FMR) is the ratio of total fatCSA to muscleCSA, as a percentage. BMR and FMR are determined at the 66 % scan site in the lower leg and the 25 % scan site in the thigh.

### Muscle area

A muscle threshold is used to create a muscle template for soft tissue analysis. Muscle area result to be compromised in many inflammatory conditions affecting childhood.

**Fig. 2:**
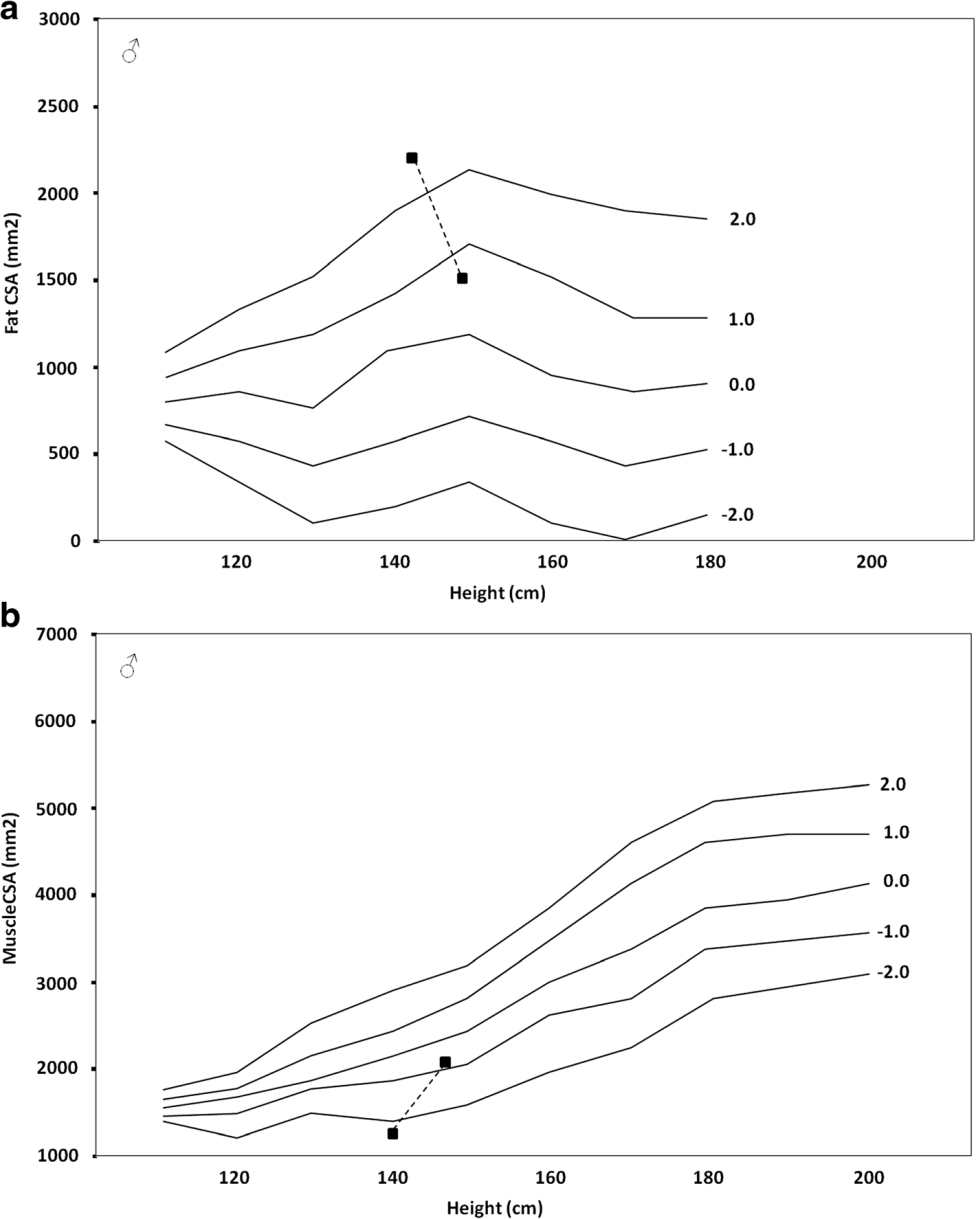
Reduction of fat CSA (fatCSA) (**a**) and increase of muscle CSA (muscleCSA) (**b**) in a boy with systemic onset juvenile idiopathic arthritis at the diagnosis and during treatment with biological drugs

Children and adolescents with JIA have decreased skeletal size, muscle mass, trabecular bone density and cortical bone geometry and strength [49]. Not surprisingly, these bone abnormalities are more pronounced in children with greater disease severity [49]. Although the muscleCSA in the lower leg was reduced in all JIA subtypes, whole-body lean mass (LBM) was diminished only in patients with systemic JIA as described in one of our previous papers [14] where muscleCSA loss ranged from 0.46 to 0.89 SD (Fig. 2), thus being not as dramatic as reported by Roth et al [50]: they found height-adjusted forearm muscleCSA measured by pQCT to be reduced by >1.25 SD in 57 patients with JIA ages 6 to 23 years, when compared with healthy controls [49]. This difference may reflect variation in joint inflammation sites and number, greater weight-bearing activity in lower versus upper extremities, or differences in our control population [49]. A linear relationship between muscle mass and bone mass is well documented in healthy children and adults [51]. In general, the greater the LBM, the higher load the muscle exerts on the bone.

### Fat area

A fat threshold is used to create a fat template within the limb area. In a cohort of pre-pubertal, free-living children, in whom DXA and pQCT measurements were acquired, the total fat mass, adjusted for lean mass, resulted positively associated with bone size, but negatively with volumetric bone density assessed by pQCT at tibia [52].

In a group of paediatric renal transplant recipients undergoing tibia pQCT assessment, muscle and fat area Z-scores were significantly lower compared with reference participants at transplantation time (both *p* < 0.01), and increased significantly within transplant recipients over the course of 12 months (both *p* < 0.001) of about 0.56 and 1.29 SD, respectively. Twelve months after transplantation, muscle area Z-scores and cortical BMD did not differ between transplant recipients and reference participants, whereas fat area Z-scores were significantly higher in transplant recipients [53].

The multivariable quasi-least squares (QLS) regression model for changes in muscle area Z-scores showed that greater increases in fat area Z-scores over each interval (*P* < 0.001) were significantly associated with greater increases in muscle area Z-scores. In the QLS model for changes in fat area Z-scores, greater glucocorticoid exposure was associated with significantly major increases in fat area Z-scores (*P* < 0.001). The link between fat area and BMD remains unclear.

Recent studies led researcher to hypothesize a strong relationship among leptin, metabolic state, and immunological self-tolerance [54]. Nutritional status, through the secretion of leptin, one of the most abundant adipocyte-derived hormones, can control immune self-tolerance, setting the basis for an exaggerated immune response to altered self or non-self, leading to chronic inflammation, metabolic dysregulation, and autoimmunity in subjects with risk factors (i.e. genetic predisposition, environment, sex, infectious agents, etc) [54, 55]. Moreover, chronic systemic inflammation, in Juvenile systemic lupus erythematosus (JSLE) patients, has shown to increase lipogenesis in nonadipose tissues and lipolysis in white adipose tissue, resulting in ectopic lipid deposition in nonadipose tissues, like muscle and liver [56] (Fig. 2). The production of proinflammatory cytokines characterizing an autoimmune disorder like systemic lupus erythematosus (SLE) could be associated with both an increased secretion of leptin and ectopic lipid accumulation in skeletal muscles, and with a higher bone turnover and consequent loss of bone mass.

### Changes in section modulus relative to changes in muscle

In order to determine if gains in muscle area after transplantation were associated with the expected gains in section modulus (i.e., the “functional muscle-bone unit”), the 12-month changes in muscle area and section modulus Z-scores were compared in the transplant recipients and a subset of 302 reference participants enrolled in an ancillary longitudinal study, adjusted for baseline muscle area and section modulus Z-scores. The latter increased with increasing muscle area Z-scores in both transplant and reference participants. However, the increase in the section modulus Z-score for a given increase in muscle area Z-score was significantly lower in transplant recipients compared with reference participants (*p* < 0.001). This impaired response was less pronounced in participants with lower section modulus Z-scores at baseline (*p* < 0.01) [53].

In recent years, an increase in the use of pQCT for regional soft tissue assessment deriving indices of adiposity and lean soft tissue has been reported. Consequently, establishment of the relationship between pQCT-derived adiposity of the limbs and the recognized standard of total body fat percentage by DXA would make it possible to reduce the need for exposure to both DXA and pQCT in paediatric subjects.

More recent research has focused on relating soft tissue properties to bone parameters [57]. Although pQCT is capable of regional soft tissue analysis, other techniques are usually employed to determine whole body soft tissue composition, frequently DXA, or both DXA and pQCT in the same investigation. The advantage of using pQCT is that fat, muscle and bone tissues proportions are provided for the same appendicular site, while DXA whole body scan gives the respective percentage of the three components in the whole body, leading to possible bias.

### The muscle-bone unit

Frost proposed a negative feedback system, i.e. the *mechanostat*, to explain how mechanics might influence bone mass and geometry, and postulated that structural adaptation is driven by the experienced bone strains [58, 59].

However, the mechanostat theory makes no assumptions about the nature of the mechanical forces causing bone strain. In line with Thompson’s conception that bone mass is influenced by the developing musculature [60], and based on the data obtained by Zanchetta’s group [61], Schiessl et al. [62] suggested that, except for traumata, it must be maximum muscle forces that cause the largest bone strains, primarily due to the poor lever arms against which most muscles work. Consequently, muscle and bone form a functional unit, the so-called *muscle-bone unit* [63]. Following the previous line of reasoning, a very strong relationship should exist between maximum muscle force and bone mass/geometry, and the former should be a better predictor for bone mass/geometry than any other proxy marker for maximum force (e.g. muscle cross-sectional area, volume, mass, and torque) [64].

Maximum voluntary ground reaction force (Fmv_m1LH_) assessed by mechanography, a new functional system named Multiple One-Legged Hopping (m1LH) can be measured during the landing phase of m1LH, where plantar flexor muscles are contracting eccentrically. A correlation study was provided between Fmv_m1LH_ and pQCT bone parameters [65]_,_

### Metabolism & bone

Interestingly, an inverse association has been found between fat infiltration in skeletal muscle, fasting insulin levels and insulin resistance, and bioactive androgens, independent of total adiposity [66]. The data regarding androgenic hormone effects on fat infiltration in skeletal muscle are very limited. A previous study among 54 healthy young eugonadal white men reported that testosterone administration decreases ectopic skeletal muscle fat infiltration [67], thus raising the possibility that lower androgenic hormone levels may promote increased fat infiltration in skeletal muscle and insulin resistance. However, it is also possible that increased skeletal muscle fat infiltration may have an effect on androgen metabolism and/or androgen production; this hypotheses should be tested in future longitudinal studies [66].

Primary prevention of OP consists of maximizing peak bone mass during childhood, adolescence and young adulthood, and minimizing bone loss later in life, especially after menopause for women [68]. A view on the adaptation of bone mass due to biomechanical forces suggests why amenorrhea and osteoporosis might be related in anorexic women [69]. Muscle strength and gravitation field-related forces provide the biomechanical environment of bone [58]. Muscular inactivity leads to reduced mass of muscle fibres, called *muscle atrophy* or *sarcopenia*. Bone adaptation to sarcopenia results in osteopenia, which typically facilitates located osteoporotic bone fractures [70, 71].

Parameters of bone density of the radius of the non-dominant side were measured by pQCT (XCT 900; Stratec Medizintechnik GmbH, Pforzheim, Germany). A single tomographic slice of 2.5 mm thickness was taken at the site of the radius whose distance to the medial border of the distal radial articular cartilage corresponded to 4 % of forearm length. Forearm length was measured using a caliper as the distance between the ulnar styloid processus and the olecranon. Volumetric total BMD (BMDtot), BMC and volumetric trabecular BMD (BMDtrab) were automatically analyzed from the tomographic image using the manufacturer’s software. BMDtrab was defined as the mean mineral density of the 45 % core area of the bone’s cross-section. BMC closely corresponds to the mass of mineral contained in the entire tomographic slice, therefore it is a useful parameter to represent bone mass.

### Bone pQCT parameters and physical activity

Physical activity and bone loading stimulates bone remodeling. Increased rates of intra-cortical remodeling increases porosity and decreases the overall density of cortical bone [72], so that newly remodeled bone has a lower cortical volumetric density (BMDcort) than older cortical bone tissue [73].

By using pQCT, a positive association between exercise during growing years and bone apposition and strength was reported [74, 75]. Muscular contractions during weight-bearing activities can site-specifically increase bone mass and strength in pre-pubertal or early pubertal children [76]. Some data in adolescent subjects showed significant correlation coefficients among muscle power, muscleCSA, and bone strength (SSI) [77]. However, a greater difference in geometry of humeral diaphysis, measured with pQCT, has been observed in the playing arm of tennis and squash players when the sportive activity was started at or before the menarche [78].

The influence of factors like gender and ethnic origin on the exercise-bone health correlation is suggested by some data. In fact, additional school-based physical education conducted over seven years was associated with greater tibial structure, strength, and region-specific adaptations in cortical bone mass and density distribution in girls, but not in boys [79]. This data was confirmed by a 4-year cluster randomized controlled trial involving 365 boys and 362 girls aged 8.1 ± 0.3 years, evaluating the effects of a specialist-taught physical education (PE) program on bone strength and body composition [80]. These last group, in comparison to other children receiving PE from general classroom teachers, showed similar gains in total body BMC, FM, and muscleCSA in both sexes, but a greater gain in total body LM, cortical area (CoA) and CoTh at the mid-tibia and mid-radius in girls [80]. In a cross-sectional study involving 724 adolescents using objective measures of physical activity, the highest impact levels of physical activity were positively associated with the periosteal bone mineral accrual at the femoral neck and other hip sites [81]. Interestingly, a study conducted in pubertal subjects disclosed that, in boys, sitting was negatively associated with tibial endosteal and periosteal circumference as well as bone strength mediated by lean mass. In pubertal girls, moderate to vigorous physical activity was positively associated with CoTh and the association was not mediated by lean mass [82].

Finally, examining the effects of weight-bearing physical activity on the content and volumetric properties of bone in a pre- to early pubertal South African black (*n* = 42) and white (*n* = 24) population, the data showed that in the low bone loading group, black children had greater femoral neck BMC, greater radius area and a greater tibia total area and strength than white children, suggesting a possible ethnic specific response to mechanical loading [83].

Regarding the importance of weight-bearing activities in conditioning bone strength, some studies did not find significant differences between swimmers and normo-active controls for bone strength indexes, structure or vBMD, suggesting that swimmers present similar bone strength and structure than controls [84].

### Radiation exposure

Analogously to DXA, radiation exposure from pQCT is low (0.01 mSv), although slightly higher if compared with DXA (0.004–0.005 mSv). [85]. PQCT radiation emission must be calibrated by the daily scanning of a fantom.

### Paediatric pQCT reference values

PQCT utilizes 3-dimensional imaging to evaluate bone geometry and true volumetric density for cortical and trabecular bone separately, making it ideal for evaluation of bone in growing children and adolescents [86].

However, as pQCT is available in a limited number of institutions, which use it mainly for research purposes, some researcher groups created normative values, even if it should be applied only in the centres where the data have been developed [87, 88].

Thus, some problems in the compilation and comparison of the data can occur, as the measuring technique, the equipment, and the analysing software may often differ considerably. Moreover, many studies reported small population sampling, frequently not representative or applicable to all age groups and/or ethnicities, making patently difficult the generalization of the results [87, 88].

A few studies reported pQCT reference data in children and adolescents. Ashby et al [89] studied 499 white children and adolescents aged 6–19 years from United Kingdom using a Stratec XCT 2000. In this study the authors produced reference charts with 25^th^, 50^th^, 75^th^, and 95^th^ centiles for males and females by age for BMDtot, BMDtrab, and bone CSA measured at 4 % of radius, and CoTh, cortical mineral content, axial moment of inertia, and bone CSA measured at 50 % of radius.

However, a study from United States [82] using a Stratec XCT 2000, evaluated 231 children, adolescents and young adults aged 5–22 years, mostly Caucasian, at the 20 % tibial site for CoA, cortical density, periosteal circumference, and endosteal circumference. Another study [90] employing a Stratec XCT 2000, and including 416 children aged 5–18 years, measured at the 4 % tibial site for BMDtrab and at the 66 % site for the total, cortical and marrow CSA, cortical BMC, BMD, and CoTh.

In Europe, a Dutch study involving 371 white children aged 6–23 years old, produced reference values for age and for pubertal stage using a Stratec XCT 2000,Total CSA, BMDtot, and BMDtrab were measured at the 4 % radius site, while CoTh, BMC, BMD, CoA, and marrow area were obtained at the 65 % proximal radius [88]. However, another Dutch group [91] published data on 469 children, where reference values at the 65 % radius site included total CSA, cortical CSA, BMDcort, BMC, and muscleCSA.

As regards geographic differences in bone health, for a sample of 665 white and black subjects aged 5–35 years in the United States, the measurements at the 38 % tibial length for BMDcort, periosteal and endosteal circumferences, BMC, and bone area were given as reference curves in relation to age [37]. Ethnic and sex differences were reported in both metaphyseal and diaphyseal bone parameters, which were not accounted for by differences in body size or skeletal maturity [92]. Furthermore, in a study conducted in 471 13-year-old South African children, metaphyseal (4 %) radial BMDtrab was greater in black girls than their white peers, whereas bone strength index was not different. However, diaphyseal (38 %) tibial values, including total area, endosteal diameter, tibial diameter, periosteal circumference and polar strength-strain index were greater in the black than in white children. Cortical density was greater in black than in white boys, and higher in girls than in boys. CoTh was major in the white group. Lower leg muscleCSA was higher in white than in black children, as well as forearm muscleCSA was higher in white than in black boys. No difference in fatCSA area was reported between the ethnic groups [92]. These data have been confirmed also in adults black men in comparison to white and South Asian men independently from body size adjustments [93].

Thus, due to the great variability in site of measurement, age ranges and groups of subjects, most of the data reported in literature cannot be compared or standardized, according to what observed by Zemel et al. [94]

### The utility of pQCT in different conditions

The application of pQCT allowed enhancing the comprehension of age-related changes and sex differences in bone microarchitecture [95]. Over the past 10 years, many studies have been published about the use of pQCT in children and adolescent with chronic diseases potentially threatening bone health [96], such as cystic fibrosis [97–100], inflammatory bowel disease [101–105], juvenile idiopathic arthritis [106–109], cancer survivors [110–112], juvenile onset lupus erythematosus [109, 113–115], hematologic disorders [116–119], mellitus diabetes [120–123], renal diseases [46, 53, 124–131], cerebral palsy [132–135], growth hormone deficiency [136, 137], and genetic disorders and syndromes [138–149] (Table 1).

**Table 1:** Literature on clinical pQCT employment for bone status assessment in chronic diseases

Disease	Type of study	Patients	Controls	Results	References
HIV	Cross-sectional study	30 HIV-infected African–American or Hispanic on ART treatment		Markedly abnormal trabecular plate and cortical microarchitecture, and decreased whole bone stiffness	Yin MT et al., 2014 [174]
Cushing’s syndrome		30 patients with endogenous Cushing’s syndrome		Lower cortical area (CoA), lower cortical thickness (CoTh), lower cortical density and lower total vBMD.	dos Santos CV et al., 2015 [175]
JIA	Longitudinal study	245 patients (172 females, 73 males) with JIA		Lower TrabBMD, muscle CSA (muscleCSA), and SSIp. In contrast, JIA showed fatCSA significantly higher than controls. but not with ERA, Longitudinally, we did not find any difference in all JIA patients in comparison with baseline, except for the SSI value that normalized. A significant negative correlation among TrabBMD and systemic and/or intraarticular corticosteroids, and a positive correlation among TNF-α-blocking agents and TrabBMD were observed.	Stagi S et al., 2014 [14]
Cystic Fibrosis (CF)	Cross-sectional study	Thirty young adults with CF		↓ bone cross-sectional area and ↓ vBMD. Cortical and trabecular microarchitecture were compromised most notably involving the trabecular bone of the tibia. These differences translated into ↓ estimated bone strength both at the radius and tibia.	Putman MS et al., 2014 [176]
	Cross-sectional study	Twelve children with CF	Age and sex- matched controls	↓ WBBMC with larger differences at older ages. Periosteal and endosteal circumferences were smaller in CF. Positive relationships of CoA and SSI with age were attenuated with CF.	Bai W et al., 2016 [98]
	Cross-sectional study	Fifty-three children with CF		Pre-pubertal males with CF had greater trabecular vBMD and total vBMD at 4 % tibia, and greater total vBMD at 4 % radius. Pre-pubertal females with CF had greater total vBMD at 66 % tibia and radius, and cortical vBMD at the radius. At puberty, the CF cohort had less BMC at 4 % tibia, and smaller muscleCSA at 66 % tibia. Pubertal CF females had a smaller bone CSA at 4 % tibia, and lower SSI at the tibia and radius sites.	Brookes DS et al., 2015 [99]
Obesity	Cross-sectional study	18 lean and 18 obese children		In obese children, radial cortical porosity, cortical pore diameter, tibial trabecular thickness was lower, whereas trabecular number was higher.	Dimitri P et al., 2015 [177]
	Cross-sectional study	68 subjects with early-onset severe obesity	73 normal-weight controls	In men CoTh in proximal radius together with Tot CSA and SSI in diaphyseal tibia favored the obese over controls, while no difference was observed in other characteristics. In women, most bone characteristics were consistently higher in obese subjects compared with controls.	Viljakainen HT et al., 2015 [178]
Klinefelter syndrome and anorchia	Cross-sectional study	20 young adults (12 KS, 8 anorchia)		Reduced tibial CoA and CoTh. Lean mass was positively associated with tibial CoA and radial total, trabecular, and volumetric density.	[Wong SC et al., 2015] [143]
SHOX mutation carriers	Cross-sectional study	22 SHOX mutation carriers	22 controls	Despite a different bone geometry in radius and tibia, subjects with a *SHOX* mutation presented no differences in BMD or failure load compared to controls, suggesting that mutations in *SHOX* gene may affect bone microarchitecture albeit not bone strength	Frederiksen AL et al. 2014 [149]
Turner syndrome	Cross-sectional study	22 Turner girls	21 unaffected females	According to pQCT of the radius, total vBMD Z-score and trabecular vBMD Z-score were not significantly different between the TS group and controls. A significant reduction in cortical vBMD and a trend in CoTh at the proximal radius in the TS group suggests that cortical density may be reduced with sparing of trabecular bone, possibily predisposing to fracture	Holroyd CR et al., 2010 [139]
Juvenile systemic lupus erythematosus (JSLE)		56 female patients		Tb.BMD and SLICC/ACR-DI were independent risk factors for vertebral fracture. JSLE patients present bone microstructure and strength deficits, particularly at the distal radius.	Paupitz JA et al., 2016 [113]
	Longitudinal study	56 JSLE patients		JSLE subjects present a reduced peak bone mass with high risk of osteoporosis in early adulthood. JSLE patients had significantly lower TrabBMD, SSI, but not muscleCSA and CBA values, while CortBMD and fatCSA were significantly increased. At longitudinal evaluation, TrabBMD, fatCSA, but not muscleCSA and CBA, remained lower in patients, but SSI and CortBMD did not differ from controls.	Stagi S et al., 2014 [114, 115]
Hemophilia A and B	Cross-sectional study	18 patients with hemophilia A and hemophilia B ($18 year old)		Lower nondominant distal tibia and radius totalBMD, trabecular BMD, and corticalBMD. In addition, PWH had significantly lower Tb.N in the distal tibia, but not radius, and increased Tb.Sp at both tibia and radius. Ct.Th was significantly lower at both sites, but not Ct.Po.	Lee A et al., 2015 [179]
Neurofibromatosis 1	Cross-sectional study	18 children with NF1		Trabecular density was more severely compromised than cortical. Peripheral QCT-derived estimates of bone strength and resistance to bending and stress were poorer among children with NF1	[Armstrong L et al., 2013] [145]
Crohn’s disease	Cross-sectional study	65 CD patients	650 reference partecipants	pQCT in CD showed a marked reduction of trabecular vBMD and cortical dimensions at diagnosis; while the trabecular vBMD improved significantly diagnosis and treatment of CD, deficits in cortical dimensions progressed	Tsampalieros A et al., 2014 [101]
	Cross-sectional study	10 CD patients		pQCT and DXA revealed important differences in the associations with auxological and disease-related factors, indicating that the measures of spine BMD may not capture disease and treatment effects on trabecular bone in IBD patients	Werkstetter KJ et al., 2013 [102]
Hypovitaminosis D	Cross-sectional study	333 girls, 230 boys		In girls, aBMD and BMC of femoral necks, CoA, CoTh, total vBMD, and trabecular thickness were significantly correlated with 25(OH)VitD levels. In boys, aBMD of bilateral femoral necks, BMC of the dominant femoral neck, CoA, CoTh, total vBMD, trabecular vBMD, BV/TV, and trabecular separation were significantly correlated with 25(OH)Vit-D levels.	Cheung TF et al., 2016 [168]
Nephrotic Syndrome	Cross-sectional study	29 affected children	29 healthy controls	Bone CSA was greater in NS group at both the metaphysis and diaphysis (*p* = 0.014); greater endosteal and periosteal circumferences (both *p* < 0.01), resulting in reduced CoTh (*p* = 0.018), but similar cortical CSA (*p* = 0.22).	Moon RJ et al., 2014 [131]
	Cross-sectional study	56 NS children	>650 reference partecipants	GC therapy was associated with increases in CortBMD-Z, potentially related to suppressed bone formation and greater mineralization. Conversely, greater expansion of CortArea-Z was associated with declines in CortBMD-Z in NS.	Tsampalieros A et al., 2013 [128]
CKD	Cross-sectional study	88 CKD cases	>650 controls	LS and WB DXA suggested greater trabecular BMD and lower cortical BMC in CKD, consistent with pQCT results. LS-BMD-Z and TrbBMD-Z were greater in CKD, while WBBMC-Z (*p* < 0.0001) and CortBMC-Z (*p* < 0.0001) were lower than controls.	Griffin LM et al., 2012 [127]

Moreover, some longitudinal studies [105, 114, 115, 126, 150] have shown correlations between bone features assessed by pQCT and disease behaviour or treatments; this technique, besides describing structural changes over time, permits a better clinical follow-up. In literature, the only randomized placebo-controlled trial which used pQCT outcomes to evaluate bone health in paediatric chronic diseases regards children with Crohn’s disease [37].

Therefore, while many data have been produced about the impact of growth and body composition deficits on skeletal health and characteristics, a consensus on how to account for these important factors and interpret their effects is not yet available.

Below, a synthesis of retrospective studies conducted in children and adolescents with chronic diseases by using pQCT is reported.

#### Cystic fibrosis (CF)

With rising life expectancy, CF has been associated with an increased prevalence of low BMD [151, 152]. However, whereas well-recognized in adults, osteopenia and increased fracture rates due to CF are not well-characterized in paediatric age. Subjects with CF show a reduced aBMD, or BMC when adjusted for lean tissue mass (LTM, proxy for muscle) [97, 153]: while prior to puberty, BMC is not compromised for the associated LTM, however, lower BMC for LTM emerged in pubertal females [153]. Besides, we have clear evidence suggesting that bone mineral gain is reduced compared to normal accretion rates during adolescent growth, potentially leading to a suboptimal PBM [153–155]. Additionally, CF specific factors cause reduced areal BMD, such as malnutrition and vitamin D deficiency secondary to pancreatic insufficiency, immobility, corticosteroid therapy and chronic inflammation [151, 152, 156].

A study conducted in children with CF showed that they had lower weight-, height-, BMI-, and whole LBM-Z and tibial length; in CF females, after adjustment for BMI-Z-score and muscleCSA, lower total and trabecular vBMD was shown, whereas the males had lower cortical, muscleCSA, and fatCSA, and their trabecular vBMD deficit approached significance. The occurrence of these differences in CF youth highlights the importance of instituting measures to optimize peak bone mass early in the course of CF [157].

In another study, CF patients showed lower whole body BMC (WBBMC) than controls with larger differences at older ages. Periosteal and endosteal circumferences were smaller in CF. Positive relationships of CoA and bone strength with age were attenuated with CF. Children with CF have similar WBBMC relative to lean mass as controls. Cortical bone area and bone strength were less in CF group compared to controls, with greater differences in older children [98].

Finally, another study evaluating pQCT parameters in children and adolescents with CF, showed that pre-pubertal males had greater trabecular vBMD and total vBMD at 4 % tibia, and greater total vBMD at 4 % radius. Pre-pubertal females with CF had greater total vBMD at 66 % tibia and radius, and cortical vBMD at the radius. At puberty, the CF cohort had less BMC at 4 % tibia, and smaller muscleCSA at 66 % tibia. Pubertal CF females had a smaller bone CSA at 4 % tibia, and lower SSI at the tibia and radius sites. Bone strength parameters were not compromised prior to puberty in this CF cohort. At puberty, the bone phenotype changed for this CF cohort, showing several deficits compared to the controls. However, bone strength was adapting to the mechanical demands of the muscle [99].

#### Inflammatory bowel diseases

In literature we have found only four papers regarding pQCT in children with inflammatory bowel disease (IBD), mainly paediatric populations of Crohn’s disease (CD) [101, 102, 104, 105]. CD and ulcerative colitis patients present multiple risk factors responsible for an impaired bone accrual, such as poor growth, delayed pubertal development, malnutrition, cachexia [158]. Moreover, elevated inflammatory cytokines, such as tumor necrosis factor α (TNF-α) and interleukin-6 [159, 160], as well as glucocorticoid therapy impair bone formation, causing reduced BMD [161].

Most of the previous studies of BMD in IBD has been relied using DXA [162–165], and children with CD diagnosis should undergo DXA assessment of lumbar spine BMD at diagnosis, and annually thereafter [166].

Studies using pQCT in CD showed a marked reduction of trabecular vBMD and cortical dimensions at diagnosis [105]. Therefore, in these patients, following diagnosis and treatment of pediatric CD, the trabecular vBMD improved significantly, whereas deficits in cortical dimensions progressed [105]. In a recent paper, the comparison of pQCT and DXA revealed important differences in the associations with auxological and disease-related factors, stressing the fact that the measures of spine BMD may not capture disease and treatment effects on trabecular bone in IBD patients [167].

#### Hypovitaminosis D

Vitamin D (25(OH)D) deficiency and insufficiency are highly prevalent among children and adolescents. 25(OH)D level is significantly correlated with bone density and bone quality key parameters. Further interventional studies are warranted to define the role of vitamin D supplementation for the improvement of bone health among adolescents. Three hundred thirty-three girls and 230 boys (12–16 years old) with normal health were recruited in summer and winter separately from local schools. Serum 25(OH)D level, bone density and quality parameters by DXA and HR-pQCT, dietary calcium intake, and physical activity level were assessed. Sixty-four point seven percent and 11.4 % of subjects resulted insufficient [25(OH)D ≤ 50 nmol/L] and deficient [25(OH)D <25 nmol/L], respectively. The mean level of serum 25(OH)D in summer was significantly higher than that in winter, without obvious gender difference. In girls, aBMD and BMC of bilateral femoral necks, CoA, CoTh, total vBMD, and trabecular thickness were significantly correlated with 25(OH)D levels. In boys, aBMD of bilateral femoral necks, BMC of the dominant femoral neck, CoA, CoTh, total vBMD, trabecular vBMD, BV/TV, and trabecular separation were significantly correlated with 25(OH)D levels [168].

#### Small-for-gestational-age patients

In a study evaluating with pQCT 46 preterm children (31 appropriate-for-gestational-age, AGA, and 15 small-for-gestational-age, SGA) at mean age 10.1 years and 27 years later, preterm AGA individuals had similar BMC and BMD height-adjusted Z-scores in adulthood compared to controls, whereas preterm SGA individuals had lower distal forearm BMC and BMD height-adjusted Z-scores in adulthood than both controls and preterm AGA individuals. Moreover, preterm SGA individuals had lower gain from childhood to adulthood in distal forearm BMC height-adjusted Z-scores than controls. In tibial CSA Z-score and SSI Z-score, where all measurements were lower than controls. Preterm SGA individuals are at increased risk of reaching low adult bone mass, at least partly due to a deficit in the accrual of bone mineral during growth. In our cohort, we were unable to find a similar risk in preterm AGA individuals [169].

#### Obesity

In 135 girls and 123 boys some authors have studied whether greater pre-pubertal adiposity (defined as BMI ≥ 85^th^ percentile for age and sex) was associated with subsequent timing of maturation and bone strength during adolescence. BMI was negatively associated with age of maturation. Overweight (OW) versus healthy-weight (HW) girls had significantly greater SSI, while OW versus HW boys had significantly greater bone strength at the tibia and femoral neck, but not radius. Analyses were repeated using biological age, which yielded reduced parameter estimates for girls but similar results for boys. Differences were no longer present following adjustment for lean mass in girls while differences at the tibia were sustained in boys. These findings demonstrate sex- and site-specific differences in the associations between adiposity, maturation and bone strength [170].

Interestingly, childhood overweight appear to be consistently associated with larger long bone cross-sections in both sexes. However, overweight in childhood may also lead to higher trabecular density in women and somewhat lower cortical density in men. Specific mechanisms underlying these associations are not known.

Total cross-sectional (ToA) and CoA at the distal and shaft sites and cortical (shaft CoD) and trabecular (distal TrD) bone density of the nonweight-bearing radius and weight-bearing tibia were evaluated with pQCT. Despite being taller in adolescence, the adult body height of overweight children was similar. In both sexes, childhood overweight was consistently associated with 5-10 % larger ToA at all bone sites measured in adulthood. CoA did not show such a consistent pattern. Women, who were overweight in childhood, had ~5 % denser TrD with no difference in CoD. In contrast, TrD in men who were overweight in childhood was not different, but their CoD was ~1 % lower [171]. In another study involving 302 NW and 143 OW children, at baseline, all bone measures (bone area, total BMD, bone strength index [BSI], SSI, and muscleCSA were significantly greater in OW compared with HW children, with the exception of BMDcort. Over 16 months, total BMD increased more in the OW children, SSI was low when adjusted for body fat in the OW group, suggesting that in OW children bone strength may be not adapt to body fat excess [172].

#### HIV infection

A study involving 31 perinatally HIV-infected youth aged 3–18 years, used pQCT assessing muscleCSA, total and cortical bone area, cortical BMD and thickness and SSI at the tibial shaft. Thirty and 18 participants returned at 12 and 24 months, respectively. At baseline, height and muscleCSA were reduced in HIV-infected patients. BMC Z-scores adjusted for height and lean mass were lower than controls at all sites except the lumbar spine. Bone area and SSI Z-scores were not different from zero after adjusting for tibial length and muscleCSA. In contrast, cortical BMD Z-scores were greater in HIV-infected youth. Z-scores for all bone outcomes showed positive trends over time in those subjects. Although HIV infection may be associated with bone mass deficits during growth, bone geometry and strength appear adapted to muscle force. Further, deficits in bone mass may dissipate over time in this population [150].

#### SHOX deficiency

*Short-stature homeobox* (*SHOX*) gene haploinsufficiency may cause skeletal dysplasia including Léri–Weill Dyschondrosteosis (LWD), a syndrome presenting the triad of low height, mesomelic disproportion and Madelung’s deformity of the wrist. Bone microarchitecture and estimated strength in adult SHOX mutation carriers have not been examined.

Twenty two SHOX mutation carriers’ bone status weas compared to 22 healthy subjects by DXA and pQCT. Areal BMD and T-scores showed no significant differences between cases and controls. Total radius area was smaller in cases than controls (*p* < 0.01). Radius cortical bone area (*p* = 0.01) and thickness (*p* < 0.01) as well as total density (*p* < 0.01) were higher in SHOX mutation carriers compared to controls. Radius trabecular bone area were smaller in SHOX mutation carriers. Tibia trabecular thickness was lower in cases (*p* = 0.01). These results remained significant after adjustment for differences in body height and when restricting analyses to females. There were no differences in BMD, radius and tibia cortical porosity or FEA failure load between groups. A segment of cortical bone defect was identified in the distal radius adjacent to ulna in five unrelated SHOX mutation carriers. Despite a different bone geometry in radius and tibia, subjects with a SHOX mutation presented no differences in BMD or failure load compared to controls, suggesting that mutations in SHOX gene may have an impact on bone microarchitecture albeit not bone strength [149].

#### Turner syndrome

In 22 girls with Turner syndrome (TS) pQCT of the radius showed that total vBMD Z-score and trabecular vBMD Z-score were not significantly different between the TS group and controls. A significant reduction in cortical vBMD and a trend in CoTh at the proximal radius was noted in the TS group, suggesting that cortical density may be reduced with sparing of trabecular bone, possibly predisposing to fracture [139].

#### Cushing syndrome

Whether higher production of glucocorticoids (GCs) within the physiological range may affect bone status in healthy children is unknown. Because dietary protein intake affects both bone and GCs, we examined the association of urinary measures of glucocorticoid status and cortical bone in healthy non-obese children, after assessment of protein intake. Subjects studied (*n* = 175, 87 males, aged 6 to 18 years) had two 24-h urine samples collected. After controlling for several covariates and especially urinary nitrogen (the biomarker of protein intake) cortisol secretion ∑C21 was inversely associated with all analyzed pQCT measures of bone quality. ∑C21 also predicted a higher endosteal and lower periosteal circumference, explaining both a smaller CoA and, together with lower BMD, a lower SSI. Therefore higher GC levels, even within the physiological range, appear to exert negative influences on bone modeling and remodeling during growth. Our physiological data also suggest a relevant role of cortisone as the direct source for intracrine-generated cortisol by bone cell 11beta-HSD1 [173].

#### Juvenile idiopathic arthritis (JIA)

Children with rheumatic diseases showed significantly reduced trabecular bone mineral density and total BMD in comparison to controls, whereas cortical BMD did not differ significantly between the groups. BMD did not correlate with duration of disease or steroid medication [109].

Children with JIA have decreased skeletal size, muscle mass, trabecular bone density, cortical bone geometry, and strength. Not surprisingly, these bone abnormalities are more pronounced in children with greater disease severity. In fact, in a study involving 48 children with JIA, a decreased tibia trabecular bone density, cortical bone size and strength, and muscle mass was reported. The tibia diaphysis was narrower with decreased muscle mass, but normal, size-adjusted bone mineral in all subtypes indicated a localized effect of bone JIA. Patients exposed to glucocorticoids and MTX or to glucocorticoids or MTX alone had greatly reduced trabecular density, cortical bone geometry properties, BMC, muscle mass, and bone strength [49].

Another study evaluating tibia pQCT in 101 JIA patients and 830 healthy control subjects, disclosed that muscleCSA and SSI Z-scores were significantly lower in patients with polyarticular JIA and those with SpA. Trabecular vBMD Z-scores were significantly lower in patients with polyarticular JIA, those with systemic JIA, and those with SpA. Significant predictors of musculoskeletal deficits included active arthritis in the previous 6 months (muscleCSA), temporomandibular joint disease (muscleCSA and SSI), functional disability (muscleCSA and vBMD), short stature (vBMD), infliximab exposure (vBMD), and JIA duration (SSI). The SSI was significantly reduced relative to muscleCSA in patients with JIA after adjustment for age and limb length. Marked deficits in vBMD and bone strength occur in JIA in association with severe and longstanding disease. Contrary to the findings of previous studies, bone deficits were greater than expected relative to the muscleCSA, which illustrates the importance of adjusting for age and bone length [108].

Finally, in a study evaluating 245 children, adolescents, and young adults, 166 of whom longitudinally evaluated, showed that, in comparison with controls, JIA patients presented musculoskeletal deficits, with significantly lower levels of trabecular bone mineral density (TrabBMD), muscleCSA, and SSI. In contrast, JIA showed fatCSA significantly higher than controls [14].

#### Juvenile systemic lupus erythematosus (JSLE)

In 56 female JSLE patients, reduced density and strength parameters and microarchitecture alterations of cortical and trabecular bones were observed. Patients with vertebral fracture (VF) exhibited a significant decrease in trabecular bone parameters solely at the distal radius (Total.BMD; Trabecular.BMD [TrbBMD]; bone volume (BV)/trabecular volume (TV); apparent modulus) and higher scores for disease damage (Systemic Lupus International Collaborating Clinics/American College of Rheumatology Damage Index (SLICC/ACR-DI)). A logistic regression univariate analysis revealed that TrbBMD and SLICC/ACR-DI were independent risk factors for VF, The study demonstrates bone microstructure and strength deficits in JSLE patients, particularly at the distal radius, and the association between VF and trabecular radius alterations [113].

In another study, involving fifty-six patients with JSLE (mean age 18.5 ± 5.7 years), 46 of these longitudinally revaluated and compared with age- and sex- matched healthy controls, disclosed that JSLE patients had significantly lower TrbBMD, SSI, but not muscleCSA and CBA values, while CortBMD and fatCSA were significantly increased. At longitudinal evaluation, TrbBMD, fatCSA, but not muscleCSA and CBA, remained lower in patients, but SSI and CortBMD were not significantly different between patients and controls. JSLE subjects present a reduced peak bone mass with high risk of osteoporosis in early adulthood. To reduce the risk, close monitoring of BMD, better control of disease activity, physical exercise and dietary intake of calcium and vitamin D are advocated to ameliorate the loss of bone mass. In patients with proved osteoporosis, therapeutic approaches including bisphosphonates should be considered [114]. In fact, in a recent study evaluating 43 JSLE patients, 138 JIA patients and 79 controls, JSLE patients had a higher CortBMD than controls and JIA patients. However, JSLE and JIA patients had a significantly reduced TrbBMD compared to controls, with no differences between JSLE and JIA. In addition, JIA patients showed a significantly reduced muscleCSA compared to JSLE and controls. Conversely, fatCSA was significantly increased both in JIA and JSLE patients when compared to controls, with no differences between the JSLE and JIA groups. Analogous results were observed in the polar resistance to stress (SSI). On longitudinal evaluation, contrary to CortBMD, the difference between BMAD SDS, TrbBMD, muscleCSA and fatCSA remains unchanged; in JSLE patients, SSI is stable in comparison to JIA and controls without any difference between the two groups. The evaluation of bone density and structure parameters in JSLE patients highlights significant differences compared with JIA patients and controls. These data might indicate a different pathogenesis of bone damage in the two entities, and suggest a different diagnostic and therapeutic approach to improve the peak bone mass [115].

#### Hemophilia A and B

Children with haemophilia are at risk of suboptimal bone mass accrual and low BMD. We recently demonstrated that although BMD in Finnish haemophilic children was within the normal range, their whole-body BMD was significantly lower and hypercalciuria more prevalent than in controls. A case-control study aimed to assess bone structure and strength by pQCT at radius in 29 physically active haemophilic children (mean age 12.2 years) and 46 age-matched controls. Children with haemophilia had decreased total BMD Z-score at the distal radius (*P* ≤ 0.001), but increased cortical bone density at the proximal radius (*P* ≤ 0.001). Total bone area at the proximal radius was significantly lower in patients (*P* = 0.002), whereas there were no differences in cortical bone area or in polar SSI, between the patients and controls. Patients with mild to moderate haemophilia and on-demand treatment had inferior bone strength compared to those with moderate to severe haemophilia and prophylaxis. Altered skeletal development in haemophilic patients assessed in the radius, resulted in smaller bone size and higher cortical bone density. Importantly, bone strength at the radius appears equal to healthy children, therefore exercise seems to have a beneficial effect on bone health in these patients [118].

#### Nephrotic syndrome

Twenty nine children with nephrotic syndrome (NS) compared to 29 age- and sex- matched healthy controls, presented similar height, but a higher weight (*p* = 0.022) and a greater body fat percentage SDS (*p* = 0.008). Tibial trabecular and cortical vBMD were similar between the two groups but bone CSA was significantly greater in NS group at both the metaphysis and diaphysis (*p* = 0.014). Endosteal and periosteal circumferences were greater in children with NS than controls (both *p* < 0.01), resulting in reduced CoTh (*p* = 0.018), but similar cortical CSA. There were no associations between cumulative glucocorticoid (GC) exposure, duration of NS or number of relapses and any bone parameter. Tibial bone CSA was increased in children with NS, and this could be interpreted as a compensatory response to increased body weight. Defects in trabecular BMD were not identified in this cohort of children with NS [131].

A recent study aimed to examine changes in vBMD and cortical structure over 1 year in 56 NS children and to identify associations with concurrent GC exposure and growth. Tibia pQCT scans were obtained at enrolment, and at 6 and 12 months. Sex, race, and age-specific Z-scores were generated for trabecular vBMD (TrbBMD-Z), cortical vBMD (CortBMD-Z), and cortical area (CortArea-Z) based on >650 reference participants. CortArea-Z was further adjusted for tibia length-for-age Z-score. Quasi-least squares regression was used to identify determinants of changes in pQCT Z-scores. At enrolment, mean TrbBMD-Z (-0.54 ± 1.32) was significantly lower (*p* = 0.0001) and CortBMD-Z (0.73 ± 1.16, *p* < 0.0001) and CortArea-Z (0.27 ± 0.91, *p* = 0.03) significantly greater in NS versus reference participants. Fortyeight (86 %) participants were treated with GC over the study interval (median dose 0.29 mg/kg/day). On average, TrbBMD-Z and CortBMD-Z did not change significantly; however, CortArea-Z decreased (*p* = 0.003). Greater GC dose (*p* < 0.001), lesser increases in tibia length (*p* < 0.001), and lesser increases in CortArea-Z (*p* = 0.003) were independently associated with greater increases in CortBMD-Z. Greater increases in tibia length were associated with greater declines in CortArea-Z (*p* < 0.01), unlike in reference participants (interaction *p* < 0.02). Thus, GC therapy was associated with increases in CortBMD-Z, potentially related to suppressed bone formation and greater secondary mineralization. Conversely, greater growth and expansion of CortArea-Z (ie, new bone formation) were associated with declines in CortBMD-Z. Greater linear growth was associated with impaired expansion of CoA in NS [128].

In another study regarding stage 4–5 chronic kidney disease (CKD) children, LS and WB DXA and tibia pQCT scans were obtained in 88 cases and >650 healthy controls, aged 5–21 years. Sex- and race-specific Z-scores were generated for BMD and BMC by DXA, relative to age and adjusted for height Z-score (LS-BMD-Z and WBBMC-Z), and compared to pQCT TrbBMD-Z for age and CortBMC-Z for age and tibia length. LS-BMD-Z (*p* < 0.0001) and TrbBMD-Z (*p* < 0.0001) were greater in CKD, and WBBMC-Z (*p* < 0.0001) and CortBMC-Z (*p* < 0.0001) were lower, compared to controls. Z-scores were correlated at trabecular (LS-BMD-Z and TrbBMD-Z: R = 0.36) and cortical (WBBMC-Z and CortBMC-Z: R = 0.64) sites in CKD, similarly to controls. LS and WB DXA suggested greater trabecular BMD and lower cortical BMC in CKD, consistent with pQCT results; however, correlations were modest. Studies are needed to identify methods that predict fracture in childhood CKD [127].

## Conclusions

The employment of pQCT has been shown to allow an accurate assessment and follow-up of bone status in several chronic inflammatory diseases in developmental age (see Table 1) [180]. Due to its negligible radiation exposure and its ability to characterize appendicular bone geometry, compartmental density and strength, and to study fat and muscle composition of limbs, as schematized in Table 2, pQCT, may represent a valid functional approach to monitor the beneficial and harmful effects of drug therapies, diet and exercise, as well as the disease outcome on bone health.

**Table 2:** Pros and cons of pQCT for bone health evaluation in developmental age [180]

Advantages of pQCT	Disadvantages of pQCT
Measure of bone geometry : marrow and cortical Cross-Sectional Area (CSA), Cortical Thickness (CoTh), periosteal and endosteal circumference.	Evaluation of only appendicular bone with low turnover (radius and tibia).
Distinct obtainment of Trabecular and Cortical BMD values	Low spatial resolution.
Good accurancy and precision.	Exact repositioning of the extremity required in follow-up.
Low radiation dose (≈3 μSv per scan), especially important in children.	Scarce data available about pQCT –based fracture risk.
Functional evaluation of pediatric bone diseases, through muscle and fat measures and biomechanical bone parameters provided.	

## Abbreviations

%BF: Percent body fat (%)
aBMD: Areal bone mineral density
AD-SoS: Amplitude-dependent speed of sound
BMAD: Bone mineral apparent density
BMD: Bone mineral density
BMI: Body mass index
BTT: Bone transmission time
CBA: Cortical bone area
CortBMD: Cortical bone mineral density
DXA: Dual-energy X-ray absorptiometry
ERA: Enthesitis-related arthritis
fatCSA: fat cross-sectional area
JIA: Juvenile idiopathic arthritis
muscleCSA: muscle cross-sectional area
PBM: Peak bone mass
pQCT: Peripheral quantitative computed tomography
QUS: Quantitative ultrasonography
SDS: Standard deviation scores
SSI: Density-weighted polar section modulus
TotBMD: Total bone mineral density
TrbBMD: Trabecular bone mineral density
vBMD: Volumetric BMD
